# Gender norms and economic empowerment intervention to reduce intimate partner violence against women in rural Côte d’Ivoire: a randomized controlled pilot study

**DOI:** 10.1186/1472-698X-13-46

**Published:** 2013-11-01

**Authors:** Jhumka Gupta, Kathryn L Falb, Heidi Lehmann, Denise Kpebo, Ziming Xuan, Mazeda Hossain, Cathy Zimmerman, Charlotte Watts, Jeannie Annan

**Affiliations:** 1 Department of Chronic Disease Epidemiology, Division of Social and Behavioral Sciences, Yale School of Public Health, New Haven, CT 06520, USA; 2 Center for Interdisciplinary Research on AIDS, Yale University, New Haven, CT, USA; 3 International Rescue Committee, New York, NY, USA; 4 Innovations for Poverty Action, Abidjan, Côte d’Ivoire; 5 Community Health Sciences, Boston University School of Public Health, Boston, MA, USA; 6 Department of Global Health & Development, London School of Hygiene & Tropical Medicine, London, UK

**Keywords:** Gender-based violence, Randomized controlled trial, West Africa, Economic empowerment, Evaluation

## Abstract

**Background:**

Gender-based violence against women, including intimate partner violence (IPV), is a pervasive health and human rights concern. However, relatively little intervention research has been conducted on how to reduce IPV in settings impacted by conflict. The current study reports on the evaluation of the incremental impact of adding “gender dialogue groups” to an economic empowerment group savings program on levels of IPV. This study took place in north and northwestern rural Côte d’Ivoire.

**Methods:**

Between 2010 and 2012, we conducted a two-armed, non-blinded randomized-controlled trial (RCT) comparing group savings only (control) to “gender dialogue groups” added to group savings (treatment). The gender dialogue group consisted of eight sessions that targeted women and their male partner. Eligible Ivorian women (18+ years, no prior experience with group savings) were invited to participate. 934 out of 981 (95.2%) partnered women completed baseline and endline data collection. The primary trial outcome measure was an overall measure of past-year physical and/or sexual IPV. Past year physical IPV, sexual IPV, and economic abuse were also separately assessed, as were attitudes towards justification of wife beating and a woman’s ability to refuse sex with her husband.

**Results:**

Intent to treat analyses revealed that compared to groups savings alone, the addition of gender dialogue groups resulted in a slightly lower odds of reporting past year physical and/or sexual IPV (OR: 0.92; 95% CI: 0.58, 1.47; not statistically significant). Reductions in reporting of physical IPV and sexual IPV were also observed (not statistically significant). Women in the treatment group were significantly less likely to report economic abuse than control group counterparts (OR = 0.39; 95% CI: 0.25, 0.60, p < .0001). Acceptance of wife beating was significantly reduced among the treatment group (β = -0.97; 95% CI: -1.67, -0.28, p = 0.006), while attitudes towards refusal of sex did not significantly change Per protocol analysis suggests that compared to control women, treatment women attending more than 75% of intervention sessions with their male partner were less likely to report physical IPV (a OR: 0.45; 95% CI: 0.21, 0.94; p = .04) and report fewer justifications for wife beating (adjusted β = -1.14; 95% CI: -2.01, -0.28, p = 0.01) ; and both low and high adherent women reported significantly decreased economic abuse (a OR: 0.31; 95% CI: 0.18, 0.52, p < 0.0001; a OR: 0.47; 95% CI: 0.27, 0.81, p = 01, respectively). No significant reductions were observed for physical and/or sexual IPV, or sexual IPV alone.

**Conclusions:**

Results from this pilot RCT suggest the importance of addressing household gender inequities alongside economic programming, because this type of combined intervention has potential to reduce levels of IPV. Additional large-scale intervention research is needed to replicate these findings.

**Trial registration:**

Registration Number: NCT01629472.

## Background

There is a paucity of evidence on effective strategies to reduce intimate partner violence (IPV) against women in conflict-affected settings. In such contexts, the prevention of gender-based violence (GBV) has not been a policy priority, and the few research and programmatic efforts pertaining to GBV have primarily focused on sexual violence perpetrated by armed groups (i.e. rape as a weapon of war ) without adequate attention to violence by intimate partners [1]. However, recent research suggests that IPV may be of greater prevalence than war-related violence victimization [1-3]. Moreover, programmatic data from the International Rescue Committee (IRC), an international humanitarian organization dedicated to addressing GBV in conflict-affected regions, show that 63% of West African women assisted by IRC for violence, sought help for violence committed by an intimate partner [4]. Evidence-based approaches to reduce IPV victimization among conflict-affected women are critical given women’s particular vulnerability to IPV, [5] the potential long-term physical and mental health sequelae, which have been well-documented, [6,7] and the likely hindrances IPV poses to achieving the Millennium Development Goals [8].

To reduce IPV and mitigate its deleterious health, economic, and social risks, economic empowerment strategies (e.g. group savings, livelihood efforts or microfinance) aiming to enable women to generate and save money have received substantial attention in development and health sectors. However, these approaches have been critiqued for their narrow focus on altering economic structures without addressing the larger gender norms that perpetuate gender inequalities and IPV [9,10]. Concerns are commonly voiced about microcredit programs that are implemented in the absence of any broader attempts to change the gendered views of male partners and the potential for increases in IPV, particularly as women become more financially empowered and more willing to challenge household gender norms [11]. Past evaluation studies of economic empowerment programs have yielded somewhat conflicting results, with programs pointing to either increased protection from IPV or increased risk of violence among women participants in economic empowerment programs [12]. In response, efforts to combine both economic empowerment and gender equity have been recommended to reduce IPV [13,14]. The IMAGE study in rural South Africa was the first trial to evaluate the impact on violence of a combined micro-credit and participatory gender training intervention. It demonstrated a 55% reduction in levels of physical and sexual intimate partner violence, as well as reductions in levels of household poverty and improved HIV communication [15,16]. These findings indicated that delivering interventions to address a combination of structural factors (i.e. poverty and broader social norms) considered to enable and sustain IPV may be essential components of programs seeking to empower communities, change behaviors and improve women’s safety [17].

As the knowledge-base on IPV programming in low and middle income countries begins to grow, intervention research is also needed on IPV within conflict-affected settings. However, to date, there is limited understanding of the effectiveness of these socioeconomic interventions within conflict-affected communities where other structural factors, including disruption of economic systems and livelihoods, may play critical roles in women’s well-being and impact programmatic outcomes [18,19].

Côte d’Ivoire, once known as the 'jewel of West Africa’ due to its relative economic stability in this precarious region, was affected by widespread conflict from 2002–2004, and again in 2010–2011. Like other West African countries [20,21] IPV levels are high. Regional estimates indicate that as many as 47.5% of women report past year IPV, [22] and a community-based survey found that some 60% of Ivorian women reported experiencing lifetime IPV [23]. Both of these figures fall within the higher end of global estimates of lifetime IPV, which range from 15-71% [24].

The objective of the current study, a two-armed randomized controlled trial (RCT) in rural Côte d’Ivoire, was to evaluate the incremental impact on levels of IPV of adding “Gender Dialogue Groups” for women and their partners (aiming to change gender norms) to an economic empowerment program for women.

## Methods

### Design, setting, and participants

Our study, *Reduction of Gender-Based Violence Against Women in Côte d’Ivoire*, is a two-armed pilot RCT implemented between October 2010 and August 2012 in north and northwestern rural Côte d’Ivoire. The study was led by Yale School of Public Health (YSPH) in partnership with Innovations for Poverty Action (IPA) and IRC.

Thirty rural villages were selected for inclusion into the trial based on their history of not having previous experience with economic empowerment programming and their status as being a priority area for intervention by IRC, the implementing agency. Six villages were excluded due to challenges with mobilizing village leaders and participants, thus yielding a final set of 24 villages. The IRC Côte d’Ivoire field staff met with village leaders and eligible women to introduce the program and study. Women and village leaders were told that all women would receive the economic empowerment program at the same time, while half of the groups would receive an additional discussion group at an earlier time point than others (ie., a waitlist control). Women were then placed into 47 groups of 15–30 women. A baseline survey was subsequently conducted, in October 2010. All groups began economic empowerment programming activities (control) in December 2010. However, due to post-election violence that occurred after the baseline survey, randomization to receive the Gender Dialogue Group (treatment) in addition to ongoing economic empowerment activities versus continuing with economic activities only (control) was delayed until September 2011. To preserve social cohesion within villages, random assignment was done via public lottery. IRC staff held a public event in each participating village where each village chief drew the names of groups within each village that would be randomized to receive the treatment. Groups not randomly drawn during the lottery were told that they would receive the gender dialogue group upon completion of the study. An endline survey was conducted from July to August 2012. Ethical approval was obtained for all study protocols through the Yale University Human Subjects Committee (#1007007040) and Innovations for Poverty Action (506.11September-003) Human Subjects Committee. Local, Côte d’Ivoire-based approval was obtained by leadership committees of all participating villages.

### Participants

**Figure 1:**
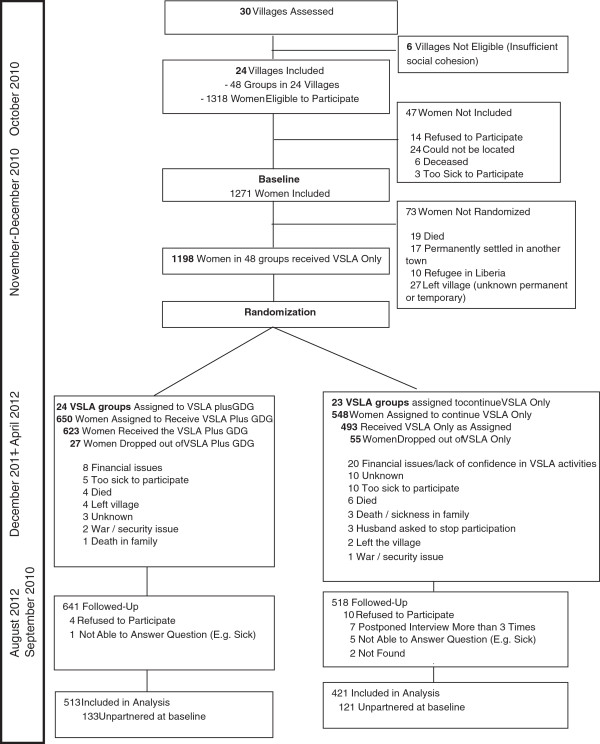
Consort diagram and timeline of study.

Eligible women were 18 and over and had no prior participation in group savings programs. Both partnered (e.g. married or in a relationship with a male for at least 1 year) and non-partnered (e.g. single, divorced, widowed) women were eligible to participate in the IRC program to preserve community social cohesion. However, non-partnered women were not considered for the IPV analytic sample. Given that the gender dialogue groups’ potential impact on IPV had not been evaluated in prior work at the time the study was being planned, effect estimates were largely unavailable. However, power calculations were conducted based on the expected number of women who would be eligible for the analytic sample per IRC projections. Power calculations revealed that the minimum detectable difference would be 13-16% at a significance level of .05 and 80% power, with a total minimum sample of 1008 eligible women. In total 1,271 women completed the baseline survey (96% response rate), of which 981 (77.2%) were partnered and thus eligible for the study. Of these 981 women, 934 (513 treatment; 421 control) were also included in the follow-up (95.2%); thus yielding the final analytic sample (CONSORT, Figure 1). Women with no children were more likely to have both missing data and drop out of the intervention; no other missingness or attrition varied by demographics or baseline IPV outcomes. Control group participants were significantly more likely to drop out of the program; IRC administrative records revealed that financial issues/lack of confidence in group savings activities was more frequently cited by control women as reasons for leaving the program (no statistical testing conducted).

### Intervention

**Figure 2:**
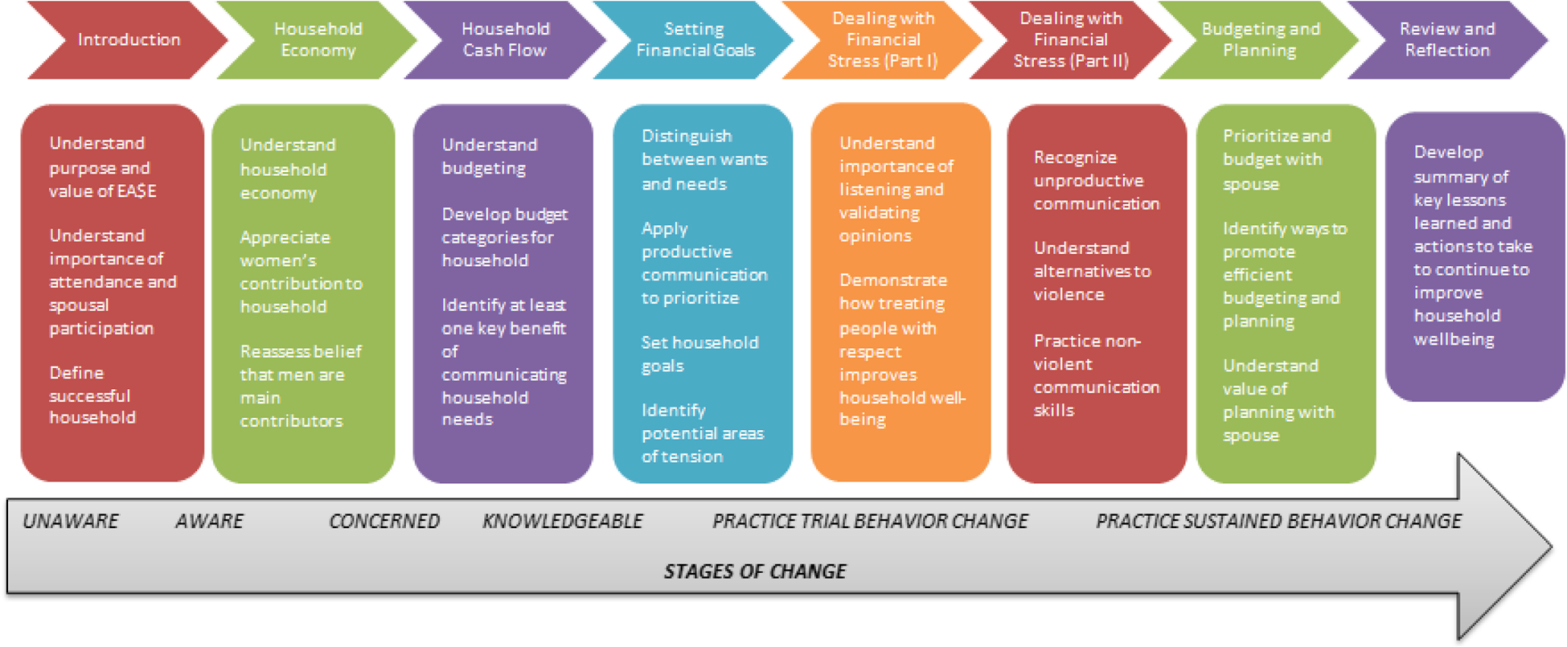
Gender dialogue group session details and the underlying theoretical assumptions.

The description of the intervention components is detailed in Table 1 and in Figure 2. To summarize, the control arm consisted of an economic empowerment group savings program (i.e. village savings and loans associations, VSLA). The treatment arm received VSLA *and* an 8-session Gender Dialogue Group (GDG), which was based on Stages of Change constructs of the Transtheoretical Model [25]. The GDGs were developed for women and their male partner and sought to address household gender inequities. The 8 GDG sessions were spread out over a 16 week period (i.e., 4 months), where meetings were held once bi-weekly. These GDG sessions met on top of the weekly VSLA sessions. Both arms met once a week for the VSLA only sessions, while groups in the treatment arm also met bi-weekly for GDG sessions. GDG sessions were designed to last between 1.5 – 2.5 hours. Sessions were facilitated by two (one male and one female) IRC field agents (one was a gender-based violence field agent while the other was an economic recovery field agent). These IRC field agents were trained on the basics of facilitation, including creating a safe and respectful environment, active listening skills, and effective questioning [26]. Each pair of facilitators was assigned to one group. Sessions typically began with a review of the previous session’s themes, discussions of the current session’s goals, and then various activities including skits, group learning exercises and discussions, and homework assignments. While topics of the GDGs varied, underscoring all sessions were messages of the importance of non-violence in the home, respect and communication between men and women, and recognition of the important contributions women make to household well-being.

**Table 1:** Description of intervention components

***Economic Empowerment Program: Village Savings and Loans Associations (VSLA)***	***Gender Dialogue Groups (GDG) Aiming to Change Gender Norms***
The VLSAs provide women with a local, safe, and convenient place to save money, access small loans, and a critical safety net in the form of an “emergency fund or social fund”. The VSLA model is simple and practical. A group of 15-30 individuals decide to save money together and contribute to a shared fund weekly. Individual members borrow from this common fund and pay the loan back at a modest interest rate, helping the fund grow over time. The group agrees on a pay-out date (generally 8-12 months after savings begins). At this time, each member receives their accumulated savings plus a percentage return on their savings. Managed appropriately, VSLAs provide affordable credit for borrowers and interest rates for savers that typically exceed those that any formal institution could provide. VSLAs employ participant-driven management which fosters sustainability, and also make this form of savings more feasible in rural regions (including conflict affected settings) lacking other formal finance institutions or where the income level of women would not allow access to financial institutions.	Participants and their male partners (or male family member if the participant does not have a male partner) were randomized to receive GDG or wait-listed until after the study was completed. GDGs create an opportunity for bringing together VSLA members and their spouses to reflect on their financial decisions and goals, the value of women in the household, and alternatives to violence. While the overall focus of GDGs is household financial well-being, each session is designed to raise underlying issues that condone IPV and challenge participants to equalize balance of power between themselves and their spouses. These discussions in turn provide an opportunity to promote women’s participation in household decision-making and encourage a shift towards more equitable spousal power relations. This approach was developed by IRC and was first piloted in Burundi in 2009 [27] Groups met every other week and took place between December 2011 and April 2012.

### Data collection

Trained local female research staff were matched to participants based on language and ethnicity. In private locations, they completed verbal informed consent with participants, verbally administered paper-based surveys and recorded respondents’ responses. Survey interviews were conducted in line with WHO ethical and safety guidelines for research on IPV [28]. Surveys were translated into French and back-translated into English. Research staff verbally translated surveys and informed consent into eleven local languages for women, as necessary. A list of local medical, legal, and psychosocial support services available for referral services was given to participants upon survey completion.

### Measures

The main outcome measure of the study was past-year physical and/or sexual intimate partner violence reported by women. For this we used the items from the WHO Multi-Country study on Women’s Health and Domestic Violence [29]. In this instrument, and as is best practice in violence research, [29] women are asked explicit questions about whether they have experienced different acts of violence. The past year prevalence of physical partner violence was assessed via whether a woman reported that her partner had slapped her or thrown something at her that could hurt her; pushed, shoved, kicked or dragged her; choked her or burned her intentionally; threatened to use a gun, knife or other weapon against her; and used a gun, knife or other weapon against her in the past year. Sexual intimate partner violence was assessed through whether women, in the past year, had been forced to have sex because of threats or intimidation or physically forced to have sex by their intimate partner when she did not want to. An affirmative response to any item was coded as experiencing past-year physical and/or sexual IPV in the final outcome variable; while “no” to all items was coded as no IPV.

Secondary outcomes included: 1) any past-year physical IPV violence; 2) any past year sexual IPV violence; 3) any past year economic abuse from their partner; and 4) gender norms. Past-year physical IPV and sexual IPV were derived from the summary measure of any physical and/or sexual IPV as described above. The secondary outcomes examine past-year sexual IPV and physical IPV independently, rather than in a summary measure of past-year physical and/or sexual IPV. Economic abuse was measured through the following three items: if in the past-year the partner: (1) had taken money against her will; (2) refused money for household necessities; or (3) obliged the woman to give him all or part of the money she earned [30]. These secondary outcomes were operationalized as a 'yes to any’/'no to all’ summary measure. Gender norms were assessed continuously via fourteen adapted items that asked if a husband was justified in beating his wife in different scenarios (Cronbach’s α = 0.91) [31]. Scenarios included if she *[the wife]* disobeys him *[the husband]*, he suspects she was unfaithful, finds out she was unfaithful, she gossips with the neighbors instead of taking care of children, she does not prepare the meals on time, she refuses to have sex with him, she does not complete her housework to his satisfaction, she neglects the children, she argues with him, she burns the food, she argues with her in-laws, she disobeys her in-laws, she does not complete her household work to her in-laws’ satisfaction, and she cannot have children. A second adapted scale, measured continuously, assessed if a woman had the right to refuse sex through eight items (if she did not want to, he is drunk, she is sick, he mistreats her, she suspects he has been unfaithful, she knows he has been unfaithful, he refuses to use condoms, she has pelvic or menstrual pain) (Cronbach’s α = 0.68) [31]. For both of the above scales, women were asked if they agreed or disagreed, and were assigned one or zero points based on their response. Women’s scores were then summed for the continuous measures. The study instrument was adapted from a questionnaire developed by researchers at the London School of Health and Tropical Medicine, [23] which was used in a complementary evaluation study in Côte d’Ivoire.

### Analysis

Data were double-entered into a Microsoft Access database [32]. The distribution of baseline socio-demographic variables were compared using Pearson chi-squared or two sample t-tests by treatment status to ascertain the results of randomization; no significant differences emerged (Table 2).

**Table 2:** Baseline characteristics of study sample, by treatment arm (N = 934)

	**Overall N = 934**^**a**^	**VSLA Only (Control n = 421)**^**a**^	**VSLA Plus GDG (Treatment n = 513)**^**a**^	**p-value**^**b**^
Age in years	37.7 (s.d. = 11.5)	37.7 (s.d. =12.1)	37.7 (s.d. = 10.9)	0.96
Marital status				
Married	767 (82.1)	342 (81.2)	425 (82.9)	
Not married	167 (17.9)	79 (18.8)	88 (17.2)	
Lives with partner	124 (13.3)	61 (14.5)	63 (12.3)	0.58
Does not live with partner	43 (4.6)	18 (4.3)	25 (4.9)	
Women’s occupation				
Farmer only	145 (15.5)	61 (14.5)	84 (16.4)	
Small business owner only	425 (45.5)	194 (46.1)	231 (45.0)	0.08
Farmer and small business owner	308 (33.0)	132 (31.4)	176 (34.3)	
Other	56 (6.0)	34 (8.1)	22 (4.3)	
Ethnicity				
Yacouba	585 (62.6)	275 (65.3)	310 (60.4)	
Senoufo, Dioula, or Guere	140 (15.0)	63 (15.0)	77 (15.0)	0.19
Other	209 (22.4)	83 (19.7)	126 (24.6)	
Education^c^				
None	657 (70.6)	288 (68.7)	369 (72.1)	
Primary	212 (22.8)	97 (23.2)	115 (21.5)	0.24
Secondary and above	62 (6.7)	34 (8.1)	28 (5.5)	
Religion^c^				
Christian	409 (44.3)	176 (42.3)	233 (46.0)	
Muslim	139 (15.1)	73 (17.6)	66 (13.0)	0.19
Traditional	161 (17.4)	67 (16.1)	94 (18.5)	
Other/None	214 (23.2)	100 (24.0)	114 (22.5)	
Number of pregnancies				
0	29 (3.1)	9 (2.1)	20 (3.9)	
1-3	220 (23.6)	111 (26.4)	109 (21.3)	0.07
≥4	685 (73.3)	301 (71.5)	384 (74.9)	
Partner’s occupation^c^				
Farming	729 (79.8)	318 (77.2)	411 (81.9)	0.08
Non-farming	185 (20.2)	94 (22.8)	91 (18.1)	

^a^Column percentages.

^b^P-value presented from chi-square or t-tests, where appropriate.

^c^Numbers do not add to 934 due to missing data.

To address clustering inherent in the data (baseline and endline outcomes were repeated measures nested within individuals that were nested within groups which were nested within villages), multilevel analysis was used to model changes in IPV by treatment status. Specifically, 4-level random intercepts models were used to evaluate the significance of the interaction term treatment status X time (e.g. baseline vs. endline) while using random effects to adjust for both autocorrelation between the two time points within individuals as well as clustering among individuals nested in groups nested in villages. The generalized mixed model in GLIMMIX procedure in SAS v9.2 [33] was used to fit the multilevel model. A significant interaction term (time X treatment status) was indicative of statistically significant differential effects of the treatment status on changes in outcomes from baseline to end-line. Odds ratios (OR), 95% confidence intervals (CI), and p-values (at the p < .05 level) were calculated to assess significance for models with binary outcomes. Betas (β), 95% CI’s, and p-values (at the p < .05 level) were computed for linear GLIMMIX models assessing continuous outcomes.

To examine the intervention effects, two types of analyses were conducted: intent to treat (ITT), and secondarily, per-protocol (PP). No covariates were included in the models for ITT analysis as randomization was successful. PP analysis utilized a 3-level intervention variable: VSLA only (comparison/referent); VSLA + GDG low adherent (where both women and their partners attended less than 75% of sessions; VSLA + GDG high adherent (where both women and their partners attended at least 75% of sessions). As the intervention was intended to target couples, adherence was determined based on the attendance of *both* women and their male partner. In the PP analysis, number of pregnancies and religion was adjusted for, as they were the only two variables statistically associated with adherence to protocol.

## Results

Demographics of participants are presented in Table 2 for the overall sample and by treatment group. No statistically significant demographic differences were found between treatment arms.

Table 3 indicates frequencies of different forms of IPV (physical and/or sexual; physical; sexual; and economic) at baseline and endline in both arms. In ITT analysis, while not reaching statistical significance, the odds of reporting physical and/or sexual IPV in the past year was lower in the VSLA + GDG in comparison to the referent (OR: 0.92; 95% CI: 0.58, 1.47, p = .72). Reductions in the likelihood of reporting of physical IPV and sexual IPV were also observed in the treatment vs. control; although the decreases did not reach statistical significance. VSLA + GDG women were significantly less likely to report economic abuse than VSLA-only counterparts (OR = 0.39; 95% CI: 0.25, 0.60, p < 0.0001). Acceptance of justification towards wife beating was significantly reduced in the VSLA + GDG group (β = -0.97; 95% CI: -1.67, -0.28, p = 0.006), while attitudes towards the ability of a woman to refuse sex did not significantly change.

**Table 3:** Distribution of study outcomes at baseline and endline, by treatment group and effect estimates of past-year intimate partner violence (Intent to Treat Analysis) (N = 934)

	**Treatment type**	**Baseline N (%)**	**Endline N (%)**	**OR† (95% CI)**	**p-value**
Physical and/or Sexual IPV	VSLA Only (Comparison)^a^	93 (22.1)	78 (21.0)	--	
	VSLA + GDG^b^	119 (23.2)	100 (20.7)	0.92 (0.58, 1.47)^i^	0.72
Physical IPV	VSLA Only (Comparison)^a^	65 (15.4)	55 (14.8)	--	
	VSLA + GDG^b^	80 (15.6)	53 (11.0)	0.69 (0.39, 1.21)^i^	0.19
Sexual IPV	VSLA Only (Comparison)^a^	44 (10.5)	53 (14.3)	--	
	VSLA + GDG^b^	71 (13.8)	68 (14.1)	0.71 (0.40, 1.25)^i^	0.24
Economic abuse	VSLA Only (Comparison)^c^	113 (27.4)	128 (34.6)	--	
	VSLA + GDG^d^	163 (32.5)	104 (21.5)	0.39 (0.25, 0.60)^j^	<0.0001
		Mean (SD)	Mean (SD)	β (95% CI)	p-value
Justification for wife beating	VSLA Only (Comparison)^e^	4.5 (4.3)	4.0 (4.0)	-0.97 (-1.66, -0.28)^k^	0.006
	VLSA + GDG^f^	4.9 (4.4)	3.4 (4.0)		
Ability to refuse sex	VSLA Only (Comparison)^g^	5.7 (1.7)	6.2 (1.5)		
	VSLA + GDG^h^	5.7 (1.8)	6.3 (1.5)	0.10 (-0.19, 0.39)^l^	0.49

†Adjusted for clustering.

^a^Total n for comparison group at baseline is 421; Total n for comparison group at endline is 371.

^b^Total n for intervention group at baseline is 513; Total n for intervention group at endline is 483.

^c^Total n for comparison group at baseline is 412; Total n for comparison group at endline is 370.

^d^Total n for intervention group at baseline is 501; Total n for intervention group at endline is 483.

^e^Total n for comparison group at baseline is 419; Total n for comparison group at endline is 401.

^f^Total n for intervention group at baseline is 511; Total n for intervention group at endline is 502.

^g^Total n for comparison group at baseline is 421; Total n for comparison group at endline is 403.

^h^Total n for intervention group at baseline is 512; Total n for intervention group at endline is 503.

^i^Total observations used in model is 1788 / 1868.

^j^Total observations used in model is 1766/1868.

^k^Total observations used in model is 1833/1868.

^l^Total observations used in model is 1839/1868.

In total, 234, or 46% of both women and men attended 75% of sessions. In PP analysis (Table 4), in comparison to VSLA only, women part of a high adherent VSLA + GDG couple were significantly less likely to report physical IPV (aOR: 0.45; 95% CI: 0.21, 0.94; p = .04). In comparison to VSLA only women, women who were part of a high adherent VSLA + GDG couple were also less likely to report the summary measure of physical and/or sexual IPV and sexual IPV, although these reductions were not statistically significant. Women who were part of low adherent couples had reduced odds of reporting physical IPV or sexual IPV and slightly increased odds of physical and/or sexual IPV, although no outcome was statistically significant. Both high and low adherent women were significantly less likely to report economic abuse in comparison to their VSLA-only counterparts (a OR: 0.47; 95% CI: 0.27, 0.81, p = 01; a OR: 0.31; 95% CI: 0.18, 0.52, p < 0.0001, respectively). Women in high adherent couples were also significantly more likely to report reduced justification of wife beating (aOR: -1.14; 95% CI: -2.01, -0.28), while the reduction in wife beating justifications for women in low adherent couples was not significant (aOR: -0.19; 95% CI: -1.13, 0.74). Attitudes toward women’s ability to refuse sex did not statistically change in either adherent group.

**Table 4:** Distribution of study outcomes at baseline and endline, by treatment group and effect estimates of past-year intimate partner violence (Per protocol Analysis) (N = 934)

	**Treatment type**	**Baseline N (%)**	**Endline N (%)**	**Adjusted OR,**^**q **^**95% CI**	**p-value**
Physical and/or Sexual IPV	VSLA Only (Comparison)^a^	93 (22.1)	78 (21.0)	--	--
	VSLA + GDG^b^ (Low adherence)	64 (22.9)	63 (24.6)	1.19 (0.69, 2.05)	0.64
	VSLA + GDG^c^ (High adherence)	55 (23.5)	37 (16.3)	0.64 (0.35, 1.16)^l^	0.14
Physical IPV	VSLA Only (Comparison)^a^	65 (15.4)	55 (14.8)	--	--
	VSLA + GDG^b^ (Low adherence)	44 (15.8)	36 (14.1)	0.93 (0.49, 1.77)	0.82
	VSLA + GDG^c^ (High adherence)	36 (15.4)	17 (7.5)	0.45 (0.21, 0.94)^l^	0.04
Sexual IPV	VSLA Only (Comparison)^a^	44 (10.5)	53 (14.3)	--	--
	VSLA + GDG^b^ (Low adherence)	38 (13.6)	41 (16.0)	0.85 (0.44, 1.64)	0.63
	VSLA + GDG^c^ (High adherence)	33 (14.1)	27 (11.9)	0.54 (0.27, 1,10)^l^	0.11
Economic abuse	VSLA Only (Comparison)^d^	113 (27.4)	128 (34.6)	--	--
	VSLA + GDG^e^ (Low adherence)	99 (36.3)	56 (21.9)	0.31 (0.18, 0.52)	<0.0001
	VSLA + GDG^f^ (High adherence)	64 (28.1)	48 (21.2)	0.47 (0.27, 0.81)^m^	0.01
		Mean (SD)	Mean (SD)	Adjusted β (95% CI)	p-value
Justification for wife beating	VSLA Only (Comparison)^g^	4.5 (4.3)	4.0 (4.0)		
	VLSA + GDG (Low adherence)^h^	5.2 (4.5)	3.9 (4.3)	-0.19 (-1.13, 0.74)^n^	0.69
	VSLA + GDG^i^ (High adherence)	4.5 (4.2)	2.9 (3.6)	-1.14 (-2.01, -0.28)	0.01
Ability to refuse sex	VSLA Only (Comparison)^j^	5.7 (1.7)	6.2 (1.5)		
	VLSA + GDG (Low adherence)^h^	5.7 (1.8)	6.3 (1.6)	0.07 (-0.32, 0.46)^o^	0.72
	VSLA + GDG (High adherence)^k^	5.7 (1.7)	6.4 (1.4)	0.12 (-0.24, 0.48)	0.50

^a^Total n for VLSA only at baseline is 416; Total n for VLSA only at endline is 368.

^b^Total n for VSLA + GDG low adherence at baseline is 275; Total n for VLSA + GDG low adherence at endline is 252.

^c^Total n for VSLA + GDG high adherence at baseline is 232; Total n for VSLA + GDG high adherence at endline is 226.

^d^Total n for VLSA only at baseline is 408; Total n for VLSA only at endline is 367.

^e^Total n for VSLA + GDG low adherence at baseline is 270; Total n for VSLA + GDG low adherence at endline is 252.

^f^Total n for VSLA + GDG high adherence at baseline is 227; Total n for VSLA + GDG high adherence at endline is 226.

^g^Total n for VLSA only at baseline is 419; Total n for VLSA only at endline is 401.

^h^Total n for VSLA + GDG low adherence at baseline is 278; Total n for VSLA + GDG low adherence at endline is 273.

^i^Total n for VSLA + GDG high adherence at baseline is 233; Total n for VSLA + GDG high adherence at endline is 229.

^j^Total n for VLSA only at baseline is 421; Total n for VLSA only at endline is 403.

^k^Total n for VSLA + GDG high adherence at baseline is 234; Total n for VSLA + GDG high adherence at endline is 230.

^l^Total observations in model is 1769/1868.

^m^Total observations in model is 1750/1868.

^n^Total observations in model is 1812/1868.

^o^Total observations in model is 1818/1868.

^p^Adjusted for categorical number of pregnancies and religion as they were statistically associated with adherence.

## Discussion

In this randomized intervention study with rural Ivorian women, the addition of a dialogue component explicitly addressing gendered social inequalities and norms alongside economic empowerment programming significantly reduced past year physical IPV among women who participated in more than 75% of the program with their male partner. The combined intervention also significantly reduced economic abuse and altered attitudes regarding the justification and acceptance of IPV among all women in the study. Encouragingly, reductions in IPV were also observed in ITT analyses, though decreases were not significant and the effect size for the overall physical and/or sexual IPV was small. Importantly, this research demonstrates that IPV reduction programs can be rigorously evaluated in a conflict-affected setting—even in the midst of a period of heightened post-election violence, and, moreover, that it is possible to observe reductions in this under-addressed yet very prevalent form of violence in such challenging and unstable contexts.

The current RCT findings are broadly consistent with the one prior South Africa-based study (the IMAGE intervention) that examined the impact of combining gender equity components with economic empowerment programming on IPV, [11,16] offering further strength to the evidence that the inclusion of gender training to socio-economic programming can offer social and health benefits, and also extending such findings to a conflict-impacted region. However, it should be noted that the current intervention possessed unique features that included activities that specifically involved male partners and the use of a financial lens with couples to talk about gendered power dynamics in the home. Moreover, the current Cote d’Ivoire intervention was a shorter intervention than the IMAGE trial intervention, which also included a larger community mobilization component.

There are also differences regarding study design. In this study, we assessed the incremental impact of adding gender equity components onto a VSLA program, while the IMAGE intervention assessed the impact of a combined micro-credit and gender training program compared with no intervention at all. In the current study, we saw significant changes among those women who were highly adherent to the intervention (or who achieved high intervention exposure), while in IMAGE, the impact was significant among all participants. Secondary analyses of IMAGE data suggest that the gender training component of the intervention was critical to its success [11]. Future research is needed to identify exact pathways of change for the current intervention. Taken together, findings illustrate the potential benefits of adding gender sensitization components onto livelihood programs for women in both conflict affected and non-conflict affected settings.

Study findings must be interpreted within the context of limitations. Firstly, as with most stigmatized health issues, IPV self-reporting may be subject to social desirability bias. Also, prior research has suggested that participants in IPV reduction interventions may over-report IPV due to increased awareness [16]. However, the directionality of such bias is difficult to determine as it is unclear whether one arm would be more or less likely to under or over report; biases away from the null would be present if over-reporting was only present in the treatment arm. Future research in post-conflict settings that includes similar quantitative assessments of IPV perpetration and gender attitudes among men would strengthen understanding of the impacts of the type of programming that was evaluated herein. Second, participants, facilitators, and researchers were not blinded to treatment status. Given that both treatment and control groups took place in the same villages, there is a chance of contamination, and thus a bias towards null findings. However, pre-study consultations with community leaders suggested that the use of a waitlist control design in large villages would minimize such threats. No crossover was observed between treatment arms per administrative records. Also, in eight villages with a smaller population, groups were only randomized to one arm due to contamination concerns. Regarding external validity, while a community-based sample was recruited, participants may differ from women who chose not to participate in the investigation.

The study was likely to be underpowered as certain villages had more widowed participants than anticipated and because of our inability to mobilize as many villages as expected, thus reducing the analytic sample. Moreover, given the relatively scarce number of interventions conducted to date that focus on the program components and the populations in the current study, findings reported herein are preliminary in nature. Additionally, since some of the villages were inaccessible during a period of post-election violence in 2010, regular fidelity monitoring of VSLA activities was not possible. However, in anticipation of possible post-election violence, the IRC team conducted training prior to the start of activities. Attendance records indicate regular meetings, including meeting at undisclosed locations during threats of intense violence. While widespread violence may have influenced the physical mobility of our study population, which may in turn have affected whether couples stayed together physically during times of insecurity, post-hoc analyses indicated that cohabitation of partners in the year preceding the endline survey did not significantly differ by treatment group, adherent group, or reporting of IPV. Although financial issues/lack of confidence in VSLA activities were frequently cited as reasons for leaving the program among those who dropped out, it is unclear if this fully explains why drop-outs differed by treatment status given that all women received VSLA simultaneously. A related potential limitation is that we were unable to assess how the success of VSLA activities influenced overall group dynamics or effectiveness of the GDGs, as the incremental effects of GDGs may be correlated with the success of the economic component of the intervention. Notably, attrition was not significantly related to IPV at baseline or endline, nor were groups with high levels of drop-outs collapsed, which minimized contamination concerns. Moreover, due to post-election violence, the start of the GDGs were delayed and could not be completed as of August 2011 as planned initially. Thus, the past year assessment of IPV is inclusive of 8 months in which the GDG was being delivered and it is unclear if the GDGs, in the context of economic empowerment programming, would have been able to reduce IPV in this shortened period. In addition, while per protocol analyses assessed adherence to the GDG sessions, we do not know which sessions were skipped. It can be argued that the sessions regarding financial stress and household economy may have more directly addressed IPV and inequitable gender norms than other sessions. Therefore, non-attendance to these sessions may have reduced the impact of the GDG component. Notably, while not all sessions were developed to explicitly discuss IPV, reports from facilitators indicate that the topic of IPV was spontaneously discussed by groups at each session. As we were able to assess adherence to the GDG intervention and impacts on summary measures of different forms of IPV, we could not examine the severity or frequency of IPV events in our data in accordance with a dose–response relationship.

These limitations notwithstanding, the current RCT has important strengths. It was done in partnership with a non-governmental organization with a long working history in Côte d’Ivoire, and incorporated specific components to maintain positive relationships with the community (e.g. inclusion of non-partnered women, non-use of a non-interventional control group due to voiced ethical concerns). Moreover, despite the use of a comparison arm that received only the economic intervention (versus a pure control), reductions in IPV and changes in attitudes towards justification of IPV were observed. All responses were prospectively assessed, and loss to follow-up was minimal despite ongoing post-election violence. Finally, although not all results reached statistical significance, the findings from both ITT and PP analyses are consistent with one another. As argued by other violence researchers and community interventionists, for complex interventions such as the one evaluated in Côte d’Ivoire, consistency and directionality of findings, in additional to statistical significance, are important components to consider [15,30,34]. The significant finding regarding improvement in attitudes towards justification of IPV is particularly important, as attitudinal changes may be proxies for norms, which may precede changes in IPV [19]; longer-term research is needed to determine this theory of change. Further, trends in IPV reduction are encouraging, given the overlap of follow-up period and delayed intervention delivery. Related to the short follow-up time, the effect size for the overall outcome measure (physical and/or sexual IPV) may have been small due to overlap between women who reported both sexual IPV and physical IPV. This overlap, combined with the possibility that it may be more feasible to influence physical IPV levels in shorter time frames compared to sexual IPV, may in part explain why effect sizes for the overall outcome (physical and/or sexual IPV) were small and did not reach significance. Longer follow-up time is needed for future work. Findings that attitudes related to the justification of physical IPV were significantly improved, but not sexual IPV further suggest the need for future research to investigate the relative difficulty of reducing sexual IPV in comparison to physical IPV. In addition, while significant reductions in sexual IPV were not found, it is possible that the GDGs may have prevented increases in sexual IPV.

## Conclusions

The current study has important programmatic implications for addressing IPV in conflict- affected settings, including areas devoid of formal financing institutions. While more research is needed to investigate the sustainability of the intervention effects in the longer term, underlying mechanisms that give rise to the observed effects, and the costs of scaling up, findings on the reduction of IPV and improvements in attitudes toward justification of IPV in the GDG + VSLA group compared to VSLA alone are promising. This research has also shown that a subtle and feasible approach to addressing IPV, such as the one employed by GDGs can also reduce women’s exposure to partner violence, even in war-affected settings where violence may be more prominent and individuals’ reluctance to discuss abuse may be exacerbated for fear of seeming divisive during community-wide attempts to maintain peace and reconciliation [35]. In addition, in conflict-affected situations, where social and financial structures might become altered and women may, out of necessity, take on what are traditionally male roles, women’s post-conflict safety may depend on fostering equitable gender norms to reduce the threat of backlash [36]. Effectively reducing IPV in combination with creating greater financial opportunities—whether organically produced or formalized through economic empowerment efforts— may lead to sustained improvements in women’s status in a post-conflict period. Moreover, while economic empowerment programs targeting women are increasing as a means to advance women’s status, health, and livelihood, these findings showcase that the addition of an intervention for women and their male partners that promotes gender equitable norms yields more reductions in IPV than economic programming alone. Results from this trial should serve as a part of an emerging evidence base to inform policy and programs on promising strategies to reduce IPV in conflict-affected settings. This type of innovative multi-sector programming is likely to translate into better health and safety [6] and greater social and economic advancements for women and their families [8].

## Competing interests

The authors have no competing interests to declare.

## Authors’ contributions

JG and JA led the conceptualization of the study. MZ, CZ, and CW contributed to the initial study conceptualization. JG, DK, JA, and KF carried out data collection and the evaluation. ZX completed statistical analyses. JG and KF led the manuscript writing. All authors have been involved in critically revising the manuscript. All authors have read and approved the final version of the manuscript. JG, principal investigator, had full access to all the data in the study and takes responsibility for the integrity of the data and the accuracy of the data analysis.

## Pre-publication history

The pre-publication history for this paper can be accessed here:

http://www.biomedcentral.com/1472-698X/13/46/prepub
